# Impact of Studying Clinical Case Notes on the Learning of Medical Students: A Comparative Study

**DOI:** 10.7759/cureus.95941

**Published:** 2025-11-02

**Authors:** Shah Jehan, Tahreem Sajjad, Babajide Obidigbo

**Affiliations:** 1 Orthopaedic Surgery, York and Scarborough Hospitals, NHS Foundation Trust, Scarborough, GBR; 2 Medicine, Barts and the London, London, GBR; 3 Orthopaedics and Trauma, London Northwest University Healthcare NHS Trust, London, GBR

**Keywords:** case-based learning, clinical case notes, experimental learning, learning outcomes, medical education, medical student assessment, medical students, real-patient exposure, surgical education

## Abstract

Objectives

The purpose of this study was to evaluate the impact of case notes on the learning of medical students. Patients’ case notes reflect real-life events and may be used as valuable learning resources for medical students. This study aims to investigate whether learning is improved when students study real case notes compared to students who do not.

Design

We allocated 14 third-year medical students into two groups during their six weeks of surgical clinical placement. Both groups received teaching on five common surgical conditions from the same clinical teaching fellow. Group 1 received standard teaching, while Group 2 received standard teaching and were also given the task of summarising clinical case notes featuring the same five conditions. Students’ knowledge was assessed at the beginning and end of their placement by a multiple-choice written paper using 25 standardised questions pertaining to the five surgical cases. The scores obtained were then analysed to establish any significant difference in the knowledge of the two groups.

Setting

The study was performed in the Epsom and St Helier University Hospitals NHS Trust. This is a tertiary care centre, affiliated with St George's, University of London.

Participants

Medical students from St George's Medical School participated in this study.

Results

There were seven students in each group. The average pre-placement score for Group 1 was 14.2, and for Group 2 it was 14. The difference between the pre-placement scores was not significant (p = 0.633). Both groups showed improvement in their scores after their placements. The average post-placement score for Group 1 was 17.1, and for Group 2 it was 19.6. We compared the assessment scores of the two groups as a measure of their improvement. The difference in their post-placement scores was significant (p =0.044).

Conclusion

The results of this study indicate that incorporating real clinical case notes into undergraduate teaching can significantly enhance students’ learning outcomes. Students who engaged with authentic patient documentation demonstrated greater improvement in knowledge compared to those who received standard teaching alone. These findings suggest that structured case note review is a valuable, practical, and easily implementable adjunct to traditional medical education, offering a bridge between theoretical learning and clinical practice.

## Introduction

Medical education requires a careful balance between the acquisition of theoretical knowledge and its practical application in clinical settings. To support this, educational strategies must be tailored from the earliest stages of training. Experiential learning, where students engage with real-life clinical situations, provides opportunities to integrate conceptual understanding with hands-on application. These approaches have been shown to enhance students’ ability to register, retain, and reproduce new information effectively [1,2].

However, experiential learning in clinical settings can be inconsistent and limited by patient availability, placement variability, and time constraints. To bridge this gap, a structured review of real patient case notes offers a complementary approach that preserves the authenticity of clinical encounters while ensuring consistent exposure to diverse conditions.

At the undergraduate level, exposure to a diverse range of clinical cases can be limited by time constraints and placement variability. Structured engagement with real patient case notes offers a potential solution. When well-prepared, these documents can mirror real patient encounters and present clinical information in a logical sequence, aiding comprehension. Organised presentation of clinical events helps students categorise and assimilate information, thereby improving memory encoding and recall [3].

Traditionally, case notes have been used for documentation, audit, and research purposes. With improved systems of clinical documentation, their potential as educational tools is increasingly recognised. Senior medical students often possess sound theoretical knowledge, yet struggle to manage patients upon entering clinical practice, largely due to limited exposure to the complexity of real-world cases. Learners typically form “illness scripts” based on textbook examples and personal experience [4,5]. However, real patients often deviate from textbook patterns, presenting with comorbidities and psychosocial elements that challenge conventional diagnostic models. Exposure to full patient narratives through case notes can introduce students to this complexity, helping refine their clinical reasoning skills.

Traditionally, case-based learning has relied on instructor-generated or textbook-derived cases, which, although useful, often simplify or idealise clinical scenarios for teaching purposes. Such artificial cases lack the complexity, uncertainty, and contextual richness of real patient documentation, limiting opportunities for learners to engage with authentic clinical reasoning processes.

A recent study among general practitioners found that reviewing patient case notes stimulated reflective learning and prompted modifications in clinical decision-making [6]. All participants endorsed case-based reviews as a valuable method of professional development. Despite this, there is limited research investigating the impact of case note-based learning in undergraduate medical education [7]. While several studies have evaluated the educational benefits of case-based learning, few have specifically examined how reviewing genuine patient case notes influences learning outcomes in medical students. Real case notes reflect the nuanced decision-making, multidisciplinary communication, and evolving patient progress that typify real-world healthcare. This gap in current literature highlights the need to explore whether studying authentic patient case notes can enhance learning more effectively than traditional teaching methods alone.

The aim of this study is to evaluate whether engagement with real patient case notes can enhance learning among medical students. We hypothesise that students who study case notes will demonstrate superior knowledge retention and reproduction compared to those who do not.

## Materials and methods

**Figure 1:**
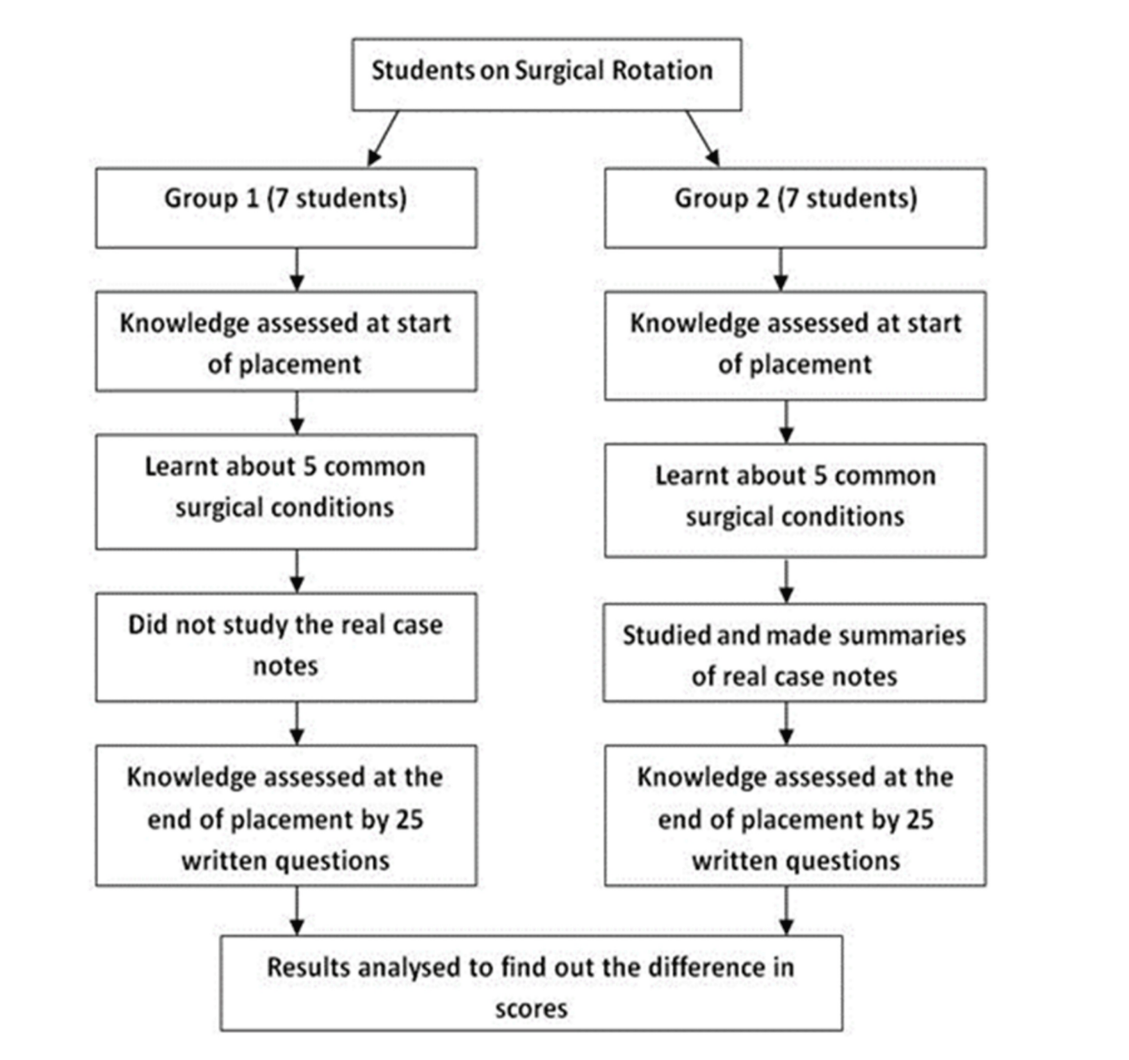
Study design

Fourteen third-year medical students participated in this study during their six-week surgical placement. The students were divided into two groups of seven, based on the pre-existing rotation schedule (Figure 1). The first rotation cohort was assigned to Group 1, and the subsequent rotation cohort was assigned to Group 2.

All students underwent a written assessment at the beginning and end of their placement, consisting of 25 standardised multiple-choice questions (MCQs). These questions assessed knowledge across five common surgical conditions: acute appendicitis, acute cholecystitis, acute pancreatitis, abdominal aortic aneurysm, and ischaemic limb. For each condition, five MCQs were developed by clinical teaching fellows, resulting in a total of 25 questions per student. The complete set of 25 MCQs is provided in Appendix A for transparency and reproducibility. The questions were designed to be appropriate for the students’ level of training and included items testing basic factual knowledge, clinical application, and ethical considerations. Question development was informed by standard surgical textbooks and examination guides, and the questions were reviewed by two senior clinicians with expertise in medical education to ensure face validity and alignment with curriculum standards. Each set of five questions per scenario addressed varied domains, including differential diagnosis, appropriate investigations, first-line management, and complications.

Group 1 received standard small-group teaching by a surgical clinical teaching fellow. The teaching fellow had four to five years of experience across multiple specialties, including general surgery, vascular surgery, orthopaedics, and intensive care, and held a Postgraduate Certificate in Biomedical Education and Membership of the Royal College of Surgeons (MRCS). The teaching sessions involved simulated cases and covered presentation, differential diagnoses, investigations, and management of the core surgical conditions. Group 1 was assessed using the same 25 MCQs in both the first and final week of their placement.

Group 2 was assessed using the same MCQ paper in the first week of placement. In addition to receiving the same standard teaching as Group 1, these students were also asked to independently study the case notes of real patients from the surgical wards. To confirm engagement with this material, students were required to produce a written summary of each of the five clinical cases. The case notes, while not standardised, followed a chronological structure and detailed the full clinical journey from admission to discharge. This included documentation of initial presentation, investigations, interdepartmental communication, operative notes (if applicable), post-operative course, and discharge planning. These notes did not include literature references or structured commentary on learning points but reflected authentic clinical documentation.

After completing their review of the case notes, Group 2 was reassessed using the same 25 MCQs. Both groups spent six weeks on their general surgical rotation and had equal contact time with the clinical teaching fellow. While Group 1 was encouraged to study textbook materials, Group 2 was instructed to study and summarise real patient case notes. However, it could not be verified whether students in Group 2 also used textbooks during their preparation.

The assessment scores of both groups were analysed to determine whether there was a statistically significant difference in performance. Data analysis, including the evaluation of pre- and post-test scores for both groups, was conducted using SPSS Statistics for Windows, version 16.0 (SPSS Inc., Chicago, Ill., USA). The Mann-Whitney U test was employed to compare changes in knowledge scores between the intervention and control groups. This non-parametric test was chosen because the small sample size and non-normal distribution of scores warranted a rank-based comparison of median differences.

This study involved human participants (medical students). Written informed consent was obtained from all participating students prior to inclusion. No identifiable patient data were collected, and therefore, patient consent was not required. The study was conducted as part of an educational evaluation within the surgical rotation and complied with institutional guidelines for student research; as such, no ethical clearance for human participants was necessary.

## Results

**Figure 2:**
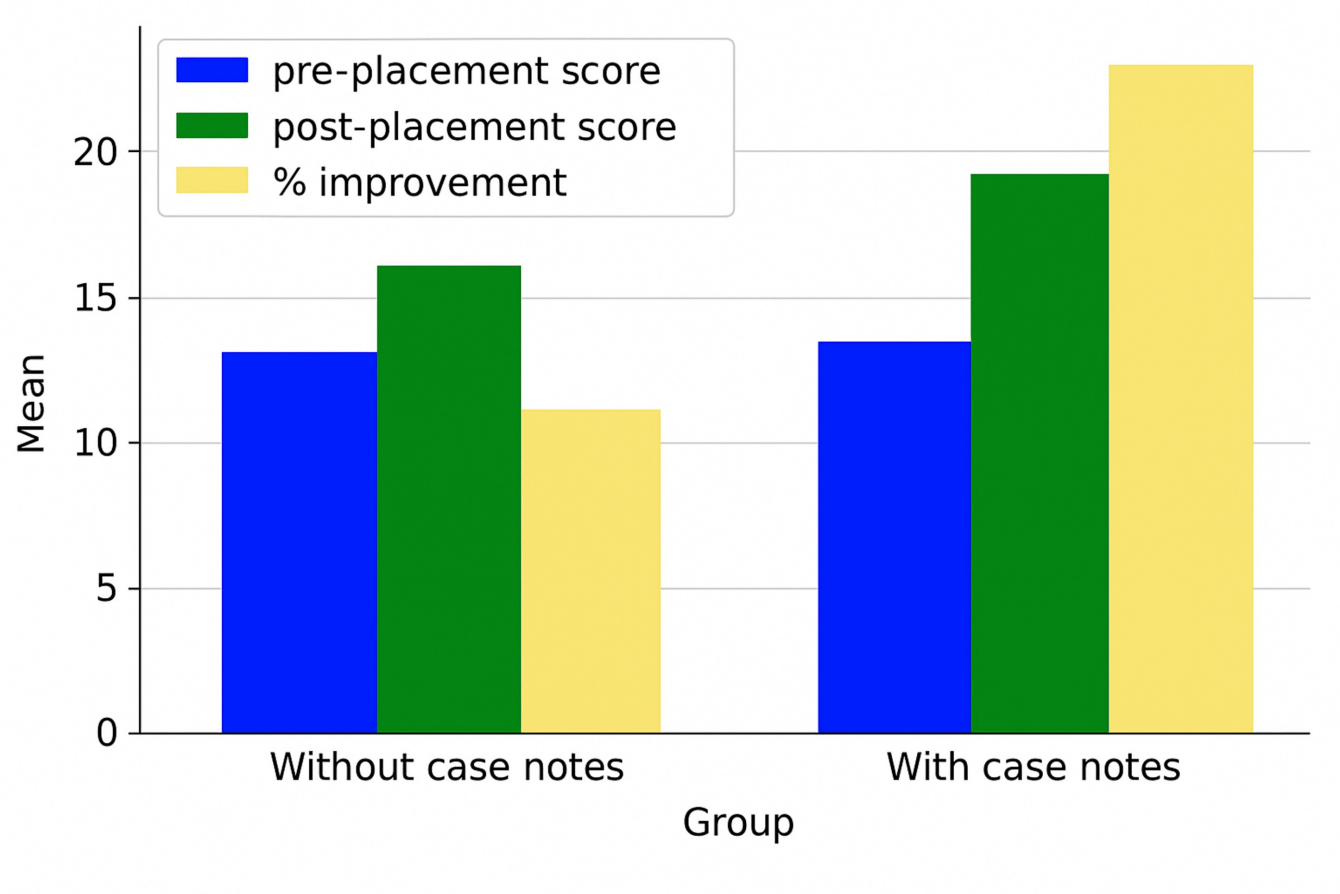
Comparison between two groups The scores at the beginning and end of the clinical attachment are shown with the percentage in improvement.

A total of 14 students were included in the study, with seven students assigned to each group. Figure 2 illustrates the comparison between Group 1 and Group 2 in terms of mean assessment scores, expressed as percentages. Table 1 provides the detailed pre- and post-placement scores for both groups.

**Table 1:** Pre- and post-placement scores for Group 1 and Group 2 Group 1 did not review case notes while Group 2 reviewed case notes of five common surgical conditions.

Group 1			Group 2		
Pre-placement	Post-placement	Improvement (% improvement)	Pre-placement	Post-placement	Improvement (% improvement)
13	16	3 (12%)	14	23	9 (36%)
15	16	1 (4%)	13	18	5 (20%)
13	18	5 (20%)	20	22	2 (8%)
13	17	4 (16%)	14	20	6 (24%)
16	17	1 (4%)	13	18	5 (20%)
17	17	0 (0%)	11	16	5 (20%)
13	19	6 (24%)	13	20	7 (28%)

In Group 1, the average pre-placement score was 14.2 ± 1.4 (range: 13-17), while the post-placement average increased to 17.1 ± 1.2 (range: 16-19). In Group 2, the average pre-placement score was 14.0 ± 3.0 (range: 11-20), rising to an average post-placement score of 19.6 ± 2.8 (range: 16-23).

Although both groups showed improvement over the six-week placement, Group 2 demonstrated a greater increase in average score following the integration of case note review into their learning process. Statistical analysis using the Mann-Whitney U test indicated that the post-placement score difference between the groups was significant (p = 0.044).

Further analysis was performed to compare the baseline knowledge between the two groups at the start of the placement. There was no statistically significant difference in pre-placement test scores between Group 1 and Group 2 (p = 0.633), suggesting that both groups began the study with comparable knowledge of the five surgical conditions.

Each group's pre- and post-placement scores were then compared to assess individual knowledge gains over the six-week rotation. Both groups demonstrated improvement in their scores, as expected, following structured teaching.

To evaluate the effect of case note review, post-placement scores were compared between the two groups. The group that engaged with clinical case notes (Group 2) showed a statistically significant improvement in post-placement scores compared to Group 1 (p = 0.044). This suggests that supplementing traditional teaching with real patient case notes may enhance knowledge acquisition among undergraduate medical students.

## Discussion

There are multiple ways to assimilate new knowledge, but effective learning is reflected in the learner’s ability to retain and reproduce knowledge when required, and to apply it flexibly across different clinical contexts [7]. In medicine, the application of knowledge is rarely straightforward. A clinician's ability to adapt depends not only on their understanding of medical theory but also on the situational context, available resources, clinical experience, and numerous patient-related factors [7].

While textbooks provide structured information on variables such as age, sex, comorbidities, and past medical history, they fall short in addressing more complex aspects of clinical care, such as communication, cultural competence, patient expectations, and psychological insight [8]. These aspects of practice are more effectively learned through real-life exposure and clinical experience [9]. Contextual learning-where students engage with patient care holistically-helps in understanding the interplay of these diverse factors and encourages integrated clinical thinking [7]. Case notes, as reflective narratives of actual patient journeys, illustrate these complexities and contextual interconnections between clinical, social, and systemic factors.

Adult learners benefit when they perceive the relevance and application of what they are learning [10,11]. Case notes support this type of experiential learning by offering a real-world stimulus. While they should not replace textbooks, they can highlight which elements of theoretical knowledge are applicable in specific clinical scenarios. In doing so, they help learners prioritise and deepen their understanding of relevant concepts.

Cox argued that clinical practice extends beyond the application of scientific knowledge, highlighting its inherently complex, multifaceted, and ambiguous nature [12]. Practising clinicians often face diagnostic, ethical, and managerial dilemmas. Case notes, when accurately and comprehensively documented, can reveal how different clinicians manage similar cases in varying ways. These discrepancies in clinical decision-making can provoke critical inquiry among learners. While answers may not be explicitly found in the notes, the questions they raise are pedagogically valuable. Educators can then use these moments to guide students through deeper learning and reflective discussion. In our study, students who reviewed case notes asked more questions, particularly around perceived discrepancies between textbook knowledge and observed clinical practice.

Despite their potential, clinical case notes are underutilised as formal teaching tools. While a few studies have acknowledged their educational value [6], there remains little published guidance on their systematic use in undergraduate training. Previous work has proposed integrating common clinical cases into pre-registration curricula, with an estimated 300 cases serving as a foundation for junior doctor training [13]. We believe this model could be adapted for undergraduate education by curating and standardising a bank of real case notes for each speciality rotation.

During clinical placements, students could complement real patient encounters with a structured review of multiple case notes. For example, a student who sees one patient with right iliac fossa pain due to appendicitis may primarily recall that diagnosis. However, reviewing five additional case notes presenting with similar symptoms but different diagnoses (e.g., ovarian torsion, mesenteric adenitis, ectopic pregnancy, Crohn’s disease, and renal colic) would encourage broader clinical reasoning. This combined exposure would better equip students to construct meaningful differentials, choose appropriate investigations, and plan context-specific management strategies.

Limitations of this study include a small sample size, with only 14 students in total, which limits the generalisability of the findings. In addition, the study assessed only short-term knowledge improvement; long-term retention of knowledge was not evaluated. Finally, although students in Group 2 were asked to focus on case notes, it cannot be excluded that they also used textbooks or other resources, which may have influenced their scores. Future studies should aim to include a larger cohort of medical students across multiple institutions and assess long-term retention to better evaluate the effectiveness of using case notes as a learning tool.

## Conclusions

This study demonstrates that reviewing real patient case notes can have a positive impact on medical students’ learning outcomes. Both groups began with comparable baseline knowledge, received identical teaching from the same clinical tutor, and followed the same curriculum. The only additional intervention for Group 2, that is, studying and summarising real clinical case notes, resulted in a significantly greater improvement in assessment scores.

Incorporating real patient case notes into undergraduate teaching can significantly enhance students’ knowledge retention and understanding compared to standard teaching methods alone. Beyond reinforcing factual knowledge, exposure to authentic documentation encourages students to appreciate the complexity of real clinical practice and to develop early clinical reasoning skills. These findings suggest that structured review of patient case notes could be feasibly integrated into existing medical curricula as a low-cost, scalable, and practical supplement to conventional teaching, helping bridge the gap between theoretical learning and clinical application.

## Appendices

Appendix A

Multiple Choice Questions Used in the Study

The following 25 multiple-choice questions (MCQs) were developed by the authors specifically for this study. Each question has one best answer.

Q1. A 19-year-old man presents with periumbilical pain that localises to the right iliac fossa. He has a low-grade fever and nausea. Which sign is most characteristic of appendicitis?

A. Murphy’s sign B. Rovsing’s sign C. Courvoisier’s sign D. Cullen’s sign E. Grey Turner’s sign

Q2. A 68-year-old man presents with sudden severe abdominal pain radiating to the back, hypotension, and a pulsatile abdominal mass. What is the most likely diagnosis?

A. Acute pancreatitis B. Ruptured abdominal aortic aneurysm C. Acute cholecystitis D. Mesenteric ischaemia E. Perforated duodenal ulcer

Q3. Which of the following is the most common cause of acute pancreatitis in the UK?

A. Trauma B. Alcohol C. Gallstones D. Hypertriglyceridaemia E. Post-ERCP (endoscopic retrograde cholangiopancreatography)

Q4. A 70-year-old man presents with an acute onset of leg pain. Examination reveals pallor, pulselessness, and paraesthesia in the limb.

What is the most likely diagnosis? A. Deep vein thrombosis B. Acute limb ischaemia C. Compartment syndrome D. Cellulitis E. Peripheral neuropathy

Q5. A 45-year-old woman presents with right upper quadrant pain, fever, and vomiting. On examination, she has right upper quadrant tenderness and inspiratory arrest on deep palpation. What is this sign called?

A. Rovsing’s sign B. Murphy’s sign C. McBurney’s sign D. Courvoisier’s sign E. Psoas sign

Q6. Which serum marker is most sensitive and specific for the diagnosis of acute pancreatitis?

A. Amylase B. Lipase C. ALP (alkaline phosphatase) D. Bilirubin E. LDH (lactate dehydrogenase)

Q7. A 65-year-old man is found to have an asymptomatic abdominal aortic aneurysm. At what diameter is elective repair generally recommended in men?

A. 3.0 cm B. 4.0 cm C. 4.5 cm D. 5.5 cm E. 6.5 cm

Q8. A 25-year-old pregnant woman (12 weeks of gestation) presents with suspected appendicitis. What is the safest first-line imaging investigation?

A. MRI abdomen B. CT abdomen C. Ultrasound abdomen D. PET (positron emission tomography) scan E. Diagnostic laparoscopy only

Q9. Which of the following is the initial management for a patient with suspected acute limb ischaemia?

A. IV antibiotics B. Immediate thrombolysis C. Intravenous heparin bolus and infusion D. Compression stockings E. Aspirin only

Q10. Which of the following is the most common complication of untreated appendicitis?

A. Intestinal obstruction B. Abscess formation C. Perforation with peritonitis D. Sepsis E. Stump appendicitis

Q11. A 40-year-old woman presents with right upper quadrant pain, fever, and jaundice. What is the most likely diagnosis?

A. Gallstone pancreatitis B. Acute cholecystitis C. Ascending cholangitis D. Viral hepatitis E. Appendicitis

Q12. Which scoring system is commonly used in the first 48 hours to assess the severity of acute pancreatitis?

A. APACHE II (Acute Physiology and Chronic Health Evaluation II) B. Glasgow-Imrie criteria C. Child-Pugh score D. Ranson’s criteria E. MELD (model for end-stage liver disease) score

Q13. A patient with a known AAA (abdominal aortic aneurysm) collapses in the emergency department. What is the immediate management priority?

A. Large-volume IV fluids to normalise BP (blood pressure) B. Transfer directly to the theatre for repair C. CT angiogram D. Endovascular repair in radiology suite E. Abdominal ultrasound

Q14. Which of the following is not one of the “6 Ps” of acute limb ischaemia?

A. Pain B. Pallor C. Pulselessness D. Petechiae E. Paralysis

Q15. Which blood test pattern is most consistent with acute cholecystitis?

A. Elevated amylase only B. Neutrophilia and raised CRP (C-reactive protein) C. Raised bilirubin and ALP without infection D. Elevated serum lipase E. Normal inflammatory markers

Q16. A 23-year-old man is diagnosed with appendicitis. What is the most appropriate definitive management?

A. IV antibiotics only B. Laparoscopic appendicectomy C. Observation and analgesia D. Colonoscopy E. Percutaneous drainage

Q17. Which complication is most feared following reperfusion of an acutely ischaemic limb?

A. Chronic pain B. Myoglobinuria leading to acute kidney injury C. Neuropathy D. Wound infection E. DVT (deep vein thrombosis)

Q18. Which sign is associated with retroperitoneal haemorrhage in severe acute pancreatitis?

A. Murphy’s sign B. Cullen’s sign C. Rovsing’s sign D. Courvoisier’s sign E. Psoas sign

Q19. A 72-year-old man with an AAA is being followed in the clinic. His aneurysm measures 5.0 cm. What is the most appropriate next step?

A. Immediate surgical repair B. Elective repair within 1 week C. Ultrasound surveillance every 3-6 months D. CT angiography E. No further follow-up required

Q20. What is the classical migration pattern of pain in acute appendicitis?

A. Right upper quadrant → epigastrium B. Periumbilical → right iliac fossa C. Epigastric → suprapubic D. Left iliac fossa → right iliac fossa E. Diffuse → left iliac fossa

Q21. Which imaging modality is most useful for diagnosing acute cholecystitis in the emergency setting?

A. Ultrasound abdomen B. CT abdomen C. MRI abdomen D. PET scan E. Plain abdominal X-ray

Q22. Which is the most serious complication of severe acute pancreatitis?

A. Pancreatic pseudocyst B. Splenic vein thrombosis C. ARDS (acute respiratory distress syndrome) D. Hypocalcaemia E. Pancreatic fistula

Q23. Which of the following is the strongest risk factor for AAA?

A. Diabetes mellitus B. Hypertension C. Smoking D. Obesity E. Hypercholesterolaemia

Q24. A patient with acute limb ischaemia has irreversible changes (Rutherford class III). What is the most appropriate management?

A. Immediate thrombolysis B. Surgical embolectomy C. Fasciotomy D. Primary amputation E. Anticoagulation only

Q25. A 36-year-old woman presents with right upper quadrant pain and fever. Ultrasound shows gallstones and a thickened gallbladder wall. What is the recommended initial management?

A. IV fluids, antibiotics, and early laparoscopic cholecystectomy B. Immediate open cholecystectomy C. ERCP D. Observation only E. Oral antibiotics and discharge
